# Physicochemical Properties and Antioxidant Activity of Spray-Dry Broccoli (*Brassica oleracea var Italica*) Stalk and Floret Juice Powders

**DOI:** 10.3390/molecules26071973

**Published:** 2021-03-31

**Authors:** María Zenaida Saavedra-Leos, César Leyva-Porras, Alberto Toxqui-Terán, Vicente Espinosa-Solis

**Affiliations:** 1 Coordinación Académica Región Altiplano, Universidad Autónoma de San Luis Potosí, Carretera Cedral Km. 5+600 Ejido San José de las Trojes, Matehuala 78700, San Luis Potosí, Mexico; zenaida.saavedra@uaslp.mx; 2 Centro de Investigación en Materiales Avanzados S.C. (CIMAV), Miguel de Cervantes No. 120, Complejo Industrial Chihuahua, Chihuahua 31136, Mexico; cesar.leyva@cimav.edu.mx; 3 Centro de Investigación en Materiales Avanzados S.C. (CIMAV), Alianza Norte No. 202, Parque de Investigación e Innovación Tecnológica (PIIT), Apodaca 66600, Nuevo Leon, Mexico; alberto.toxqui@cimav.edu.mx; 4 Coordinación Académica Región Huasteca Sur, Universidad Autónoma de San Luis Potosí. Km 5, Carretera Tamazunchale-San Martin, Tamazunchale, San Luis Potosi 79960, Mexico

**Keywords:** broccoli juice, spray drying, antioxidant activity, conservation of antioxidants, physicochemical characterization

## Abstract

This research presents the microencapsulation and conservation of antioxidants of broccoli juice processed by spray drying, and proposes the use of a by-product as a technological application. Broccoli juice (BJ) extracted from two sources, stalks and florets, was spray-dried employing maltodextrin (MX) as a carrier agent at concentrations of 5, 7.5, and 10%, and inlet temperatures of 150 and 220 °C. The total phenolic content (TPC), and antioxidant activity (AA) of the BJ-MX powders were determined together with the physicochemical characteristics, including particle morphology, microstructure, and thermal properties. Based on the TPC and AA, the optimal processing conditions found were 5% of MX and a drying temperature of 220 °C. However, the florets showed higher TPC, while stalks presented higher AA under those processing conditions. The particles exhibited micrometric sizes and a mixture of spherical-shape particles and pseudo-spherical particles. The diffractograms indicated an amorphous microstructure in all samples. The glass transition temperature (Tg) was determined in the range of 50 °C for the samples dried at 150 °C and 55 °C for those dried at 220 °C. This suggested that powders might be stored at temperatures below the Tg without presenting any loss of antioxidants.

## 1. Introduction

Broccoli cultivation is carried out throughout the year, preferably in climates with warm temperatures between 18 and 23 °C [1]. In 2019, the main producing countries of this vegetable were China and India with 73% of total production (19.7 million tons), while the United States, Spain, and Mexico each produced approximately 1 million tons [2]. Because of the growing global consciousness of healthy green lifestyles, fresh and processed broccoli consumption has rapidly grown, reaching a 940% increase [3]. The functional properties of broccoli are attributed to its bioactive compounds, which induce various functions such as antioxidant activity, enzyme regulation, apoptosis control, and cell cycles. The health-promoting components of broccoli include isothiocyanate, which is a sulfur-containing organic phytochemical compound formed after the enzymatic hydrolysis of glucosinolates [4]. Broccoli also contains vitamins such as ascorbic acid and tocopherol, minerals such as iron, zinc, and selenium, and polyphenols such as kaempferol and quercetin. These compounds are presumably responsible for reducing the risk of degenerative disorders such as cancer and cardiovascular diseases [3,4,5,6]. In addition to being a rich source of fiber, broccoli in its overall composition contains 89% water, 6.27% carbohydrates, 2.57% protein, 0.34% lipids, 0.83% ash, and 1% of the aforementioned vitamins and minerals, among others [7]. Despite the nutritional contribution of broccoli, its consumption is preferred by persons with a developed taste. It is believed that the sulfur-containing compounds impart a strong smell and bitter taste. However, these compounds have shown anticancer activity [8,9].

Due to the rapid deterioration of the vegetable under normal conditions of humidity and temperature (i.e., 50% and 25 °C), the raw vegetable must be stored at temperatures lower than ambient (about 15 °C), while the processed vegetable must be packed and frozen. In consequence, the low chemical, biological, and thermal stability under storage conditions provokes the loss of the bioactive compounds, restricting their inclusion into complex matrixes such as processed foods [10,11]. Spray drying is a practical technique for food processing that promotes the conservation of antioxidants and the encapsulation of active compounds in the form of powdered products [12]. This technique has gained popularity in segments of the food industry because of the reduced packing volume, storage, and transportation costs [13,14,15,16]. Spray drying has been employed in the encapsulation of active compounds during the drying of vegetables and fruit juices [17,18].

Broccoli has dense green edible clusters of flower buds (florets) [19]. Although the most commonly consumed organs in broccoli are the florets and the upper stems from the head [3], the leaves and stalks are considered as byproducts [20]. Nowadays, new processed foods using broccoli byproducts have attracted attention. For example, they include fresh filled pasta prepared by adding broccoli extract [21], bread with added broccoli powder [22], and gluten-free mini sponge cakes with added broccoli leaves in freeze-dried powder [23]. Several works have reported the antioxidant activity and total phenolic content of different spray-dried vegetable juices (see Table S1 from the Supplementary Data). However, regarding broccoli, most studies have reported only the antioxidant and anticancer properties of liquid extracts (see Table S2 from the Supplementary Data), while only one has evaluated the effects of drying conditions on the bioactive compounds and antioxidant activity of broccoli powder obtained in a pilot-plant tray dryer [24]. Therefore, the aim of the present work was to set the optimal processing conditions to obtain broccoli juice (BJ) powder by the spray-drying process. Low molecular-weight maltodextrin (MX) was employed as a carrier agent in order to promote the conservation of antioxidants. BJ was prepared from two different parts of the broccoli sprout (florets and stalks). The effect of the inlet temperature and MX concentration on the antioxidant activity was studied, and the physicochemical properties compared. This study contributed to obtaining BJ-MX powder and the microencapsulation of antioxidants. Technological applications include the reduction of volume, the preservation of the physical properties in powder form, the potential increase of product shelf life, and the use of byproducts such as the stalks.

## 2. Results and Discussions

### 2.1. Total Phenolic Content and Antioxidant Activity

**Figure 1:**
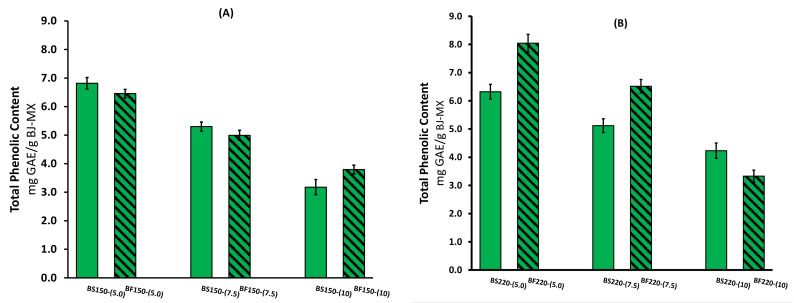
Total phenolic content of spray-dried broccoli juice–maltodextrin (BJ-MX) powders under different experimental conditions. Inlet temperature of (**A**) 150 °C, and (**B**) 220 °C.

The total phenolic content (TPC) of the BJ-MX spray-dried powders from stalks and florets is presented in Figure 1. The highest TPC value of 8.05 mg GAE/g dry wt. was obtained for BJ extracted from the florets and dried at 220 °C with 5% MX (BF220-[5.0]) content. The lowest TPC value was presented by the sample identified as BS150-[10]. Despite the drying temperature and the broccoli source, the TPC decreased with incremental increases in MX content. This behavior may be caused by a dilution effect induced by the MX, where the BJ is being replaced by the increased MX content. Obeiro and Sigo (2015) observed a similar behavior in total carotenoid content (TCC) as the content of MX varied from 3 to 10% in the drying of watermelon juice [25]. Islam et al. (2021) conducted a study using micro wet milling and spray drying to produce mandarin juice powders employing maltodextrin as a carrier agent in a concentration range of 30–70% and observed a reduction in the TPC value as the MX concentration increased [26]. From the two systems (broccoli florets and stalks), the powders obtained at higher drying temperature presented relatively higher TPC values. This is an advantageous characteristic, because in the spray-drying process, as the drying temperature increases, more BJ powder is obtained. Sarabandi et al. (2019) encapsulated eggplant peel extracts with maltodextrin, finding a TPC value of 5.03 mg GAE/g, and the value increased as the inlet temperature was elevated from 140 to 170 °C [27]. They explained that at higher temperatures, it is possible to rapidly form a protective film around the particles. Consequently, more polyphenols are encapsulated within the microstructure of the carrier agent.

However, when comparing the effect of the broccoli source, the BJ samples extracted from the florets showed relatively higher TPC values than those obtained from the stalks. Domingues-Perles et al. (2010) studied the bioactive ingredients of stalks and leaves of three varieties of broccoli by high performance liquid chromatography (HPLC) [20]. They reported a TPC value for broccoli stalks in a range of 8.12–11.74 mg/g dry wt. Among the phenolic compounds, they found that the hydroxycinnamic acids and flavonoids in the stalks of the three cultivars were in a low concentration range, while the concentration of vitamin C varied significantly among the cultivars (2.295–3.365 mg/g dry wt.). Gliszczynska-Swiglo et al. (2006) studied the effect of steam and water cooking on broccoli florets and analyzed the bioactive compounds quantified by HPLC [28]. They reported a TPC value of 8.863 mg/g dry wt. in the freeze-dried florets, while the concentration of vitamin C was in the order of 6.812 mg/g dry wt.; both studies determined a higher amount of vitamin C in the broccoli florets than in the broccoli stalks. According to Everrete et al. (2010), additionally to the phenolic compounds, the Folin–Ciocalteu reagent can react with other molecules such as ascorbic acid [29]. Thus, the differences found in the BJ-MX powder from floret and stalk juice may be related to the vitamin C content.

In contrast to this work, where only water-soluble compounds were extracted from broccoli florets and stalks, other studies have done the extraction of bioactive compounds with a methanol-water solution [5,20,28]. According to Podsedek (2007) dietary antioxidants can be divided into water-soluble antioxidants such as vitamin C and phenolic compounds such as flavonoids, and lipid-soluble antioxidants such as carotenoids and vitamin E [30]. Clearly, for food-processing purposes, it is preferable to use a solvent with low or no effect on health.

**Figure 2:**
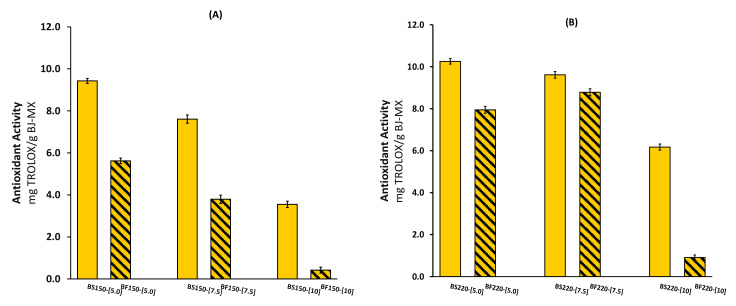
Antioxidant activity of spray-dried BJ-MX powders under different experimental conditions. Inlet temperature of (**A**) 150 °C, and (**B**) 220 °C.

The antioxidant activity of the BJ-MX powders measured by the DPPH method is presented in Figure 2. As is known, many biologically active molecules such as vitamin C, phenolic compounds, and carotenoids in plants may contribute to antioxidant capacities [31], and broccoli is not an exception [20,28,30]. The antioxidant activity presented a behavior similar to TPC with different drying temperatures and MX content. However, the effect from the broccoli source was different. The BJ-MX powders extracted from the stalks showed a higher level of antioxidant activity, suggesting that more antioxidant compounds are localized in this part of the vegetable than in the florets. The highest value was recorded for the sample extracted from the stalks with 5% MX content and dried at 220 °C (BF220-[5]). The lowest antioxidant activity was observed in the sample extracted from the florets with 10% MX and dried at 150 °C (BF150-[10]). Alvarez-Jubete et al. (2013) compared the antioxidant activity of broccoli-enriched soups and found that the floret soup showed higher activity than the stalk soup [5]. Dominguez-Perles et al. (2010) analyzed extracts of broccoli by-products and found a higher DPPH scavenging capacity in leaves than in the stalks [20]. Although vitamin C largely contributes to the antioxidant performance of many vegetables, in broccoli stalks it has been found to be in an inverse correlation with radical scavenging activity. This suggests that other molecules may contribute to radical scavenging because broccoli phenolics were directly correlated with the antioxidant DPPH test results. Duan et al. (2021) investigated the flavonoid content of 15 broccoli sources [32]. They found slight differences in the total antioxidant capacity between the leaves and florets, and explained that the antioxidant capacity was affected by the total content of flavonoids and kaempferol.

### 2.2. Thermal Characterization

**Figure 3:**
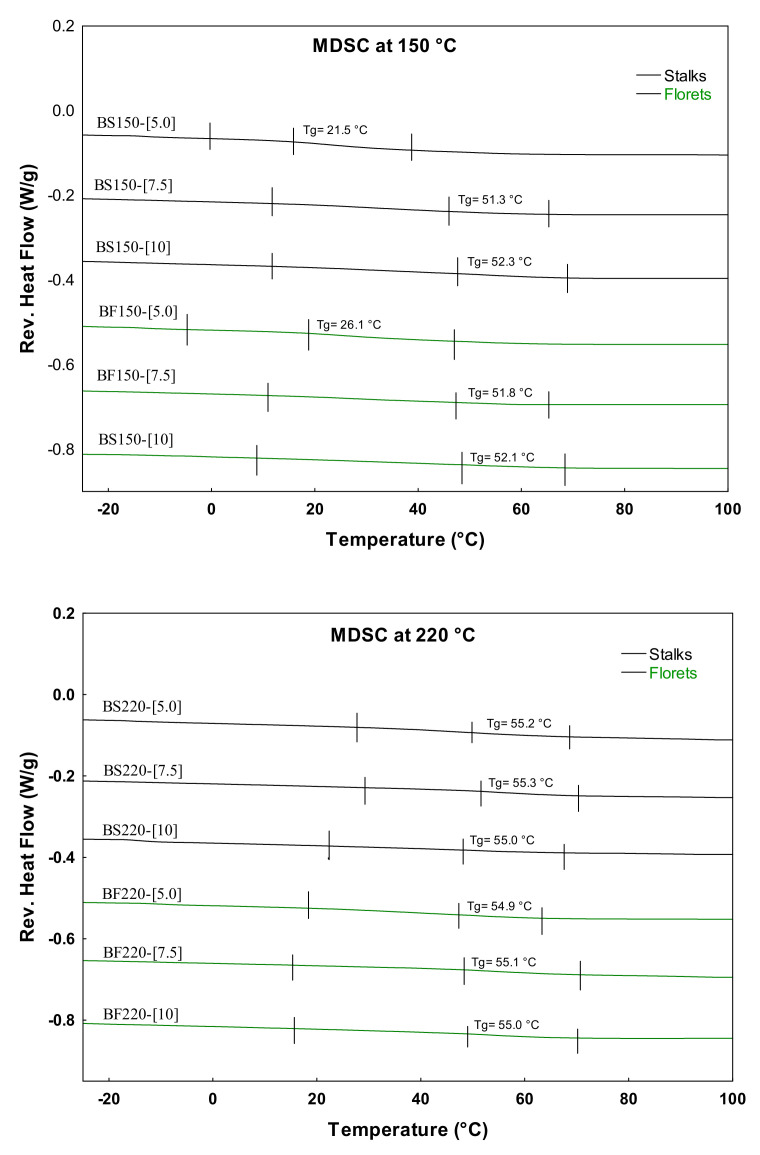
Modulated differential scanning calorimetry (MDSC) thermograms for determining the Tg of the BJ−MX powders. Tg was determined as the change in the slope of the curve.

Phase transitions promote variations in the thermal, mechanical, and diffusion properties of foods as a consequence of changes in molecular mobility associated with the transitions. Therefore, the study of phase transitions is of great importance for the control, distribution, and storage of foods. In the case of foods with low moisture content, the transitions of the main components of the food, such as proteins, carbohydrates, and lipids, play a predominant role in the food product’s physical properties. In this respect, the main solid components in the BJ-MX powder are the carbohydrate polymers (glucose molecules) contained in the maltodextrin, followed by microencapsulated antioxidants and other components in lower proportions. Thus, in order to compare the effects of the microencapsulated compounds on the thermal properties, modulated differential scanning calorimetry (MDSC) was employed to determine the glass transition temperature (Tg) of the BJ-MX powders. Figure 3 shows the section of the thermograms employed to determine the Tg of the BJ-MX powders dried at 220 and 150 °C.

The Tg values for the samples dried at 150 °C were about 51 °C, while for those samples dried at 220 °C, they were about 55 °C. Based on these values, both systems may be stable when storage is at temperatures below the Tg and relatively low atmospheric humidity. From the two systems, the powders prepared at 220 °C may present higher stability. However, the samples identified as BS150-[5.0] and BF150-[5.0] presented Tg values close to 25 °C. From these results, some valuable storage information may be inferred. For example, these two powder samples must be stored at temperatures below 25 °C, otherwise the powder may suffer a solid-state change from the amorphous state into the rubber state. In the rubber state, the microencapsulated compounds may be released from the powder particles, losing their antioxidant properties.

**Figure 4:**
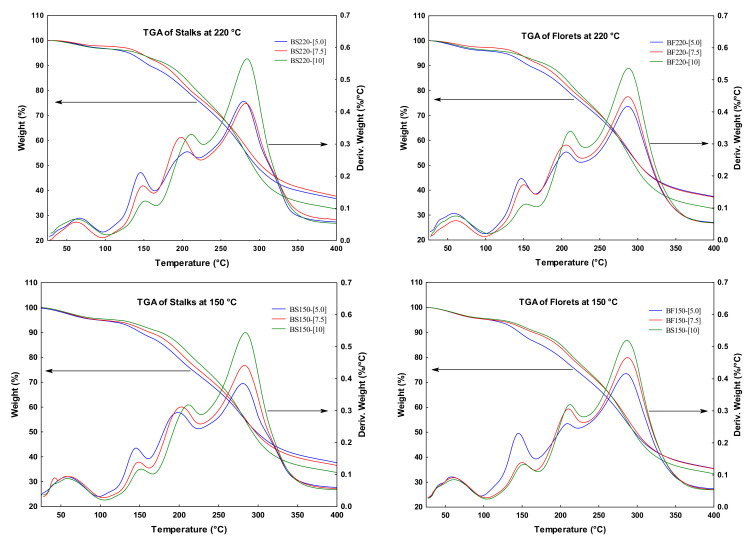
Thermogravimetric analysis of the BJ-MX powders. TGA is read on the left side of each plot, while the derivative of the weight may be read on the right side.

With the use of thermogravimetric analysis (TGA), it is possible to obtain the mass loss and its derivative as a function of temperature. Figure 4 shows the TGA curves of the BJ-MX powders. In the TGA curve, the first thermal event was observed in the temperature range of 70–110 °C, which was associated with a first-order transition such as evaporation of water, corresponding to a water loss of approximately 3.5–5%. A second and more pronounced thermal event occurred in the temperature range of 125–175 °C, accompanied by a mass loss of 15–20% that was associated with the melting of simple carbohydrates and other components of broccoli powder. A third thermal event was observed in the temperature range of 175–250 °C, corresponding to a mass loss of 20–25%, and it was related to the melting of complex carbohydrate polymers with a high molecular weight, such as those contained in maltodextrin. The last thermal event was identified in a range of 250–350 °C with a mass loss of 30–35%. This was attributed to the thermal decomposition (Td) of the long molecular chains, polymerization processes, and isomerization reactions associated with dehydration.

### 2.3. Microestructural Analysis

**Figure 5:**
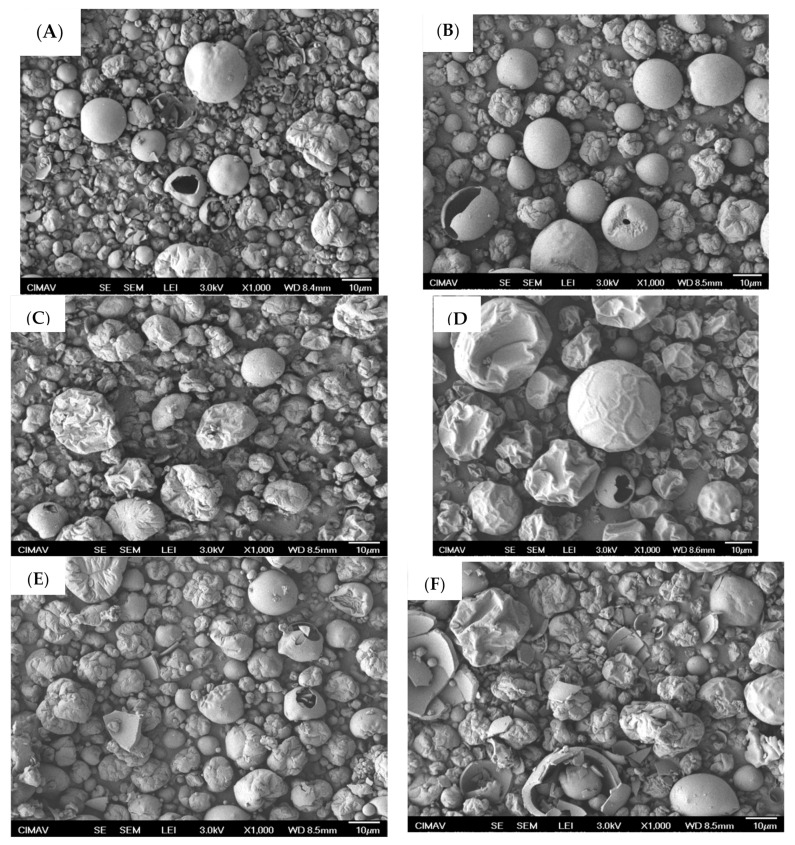

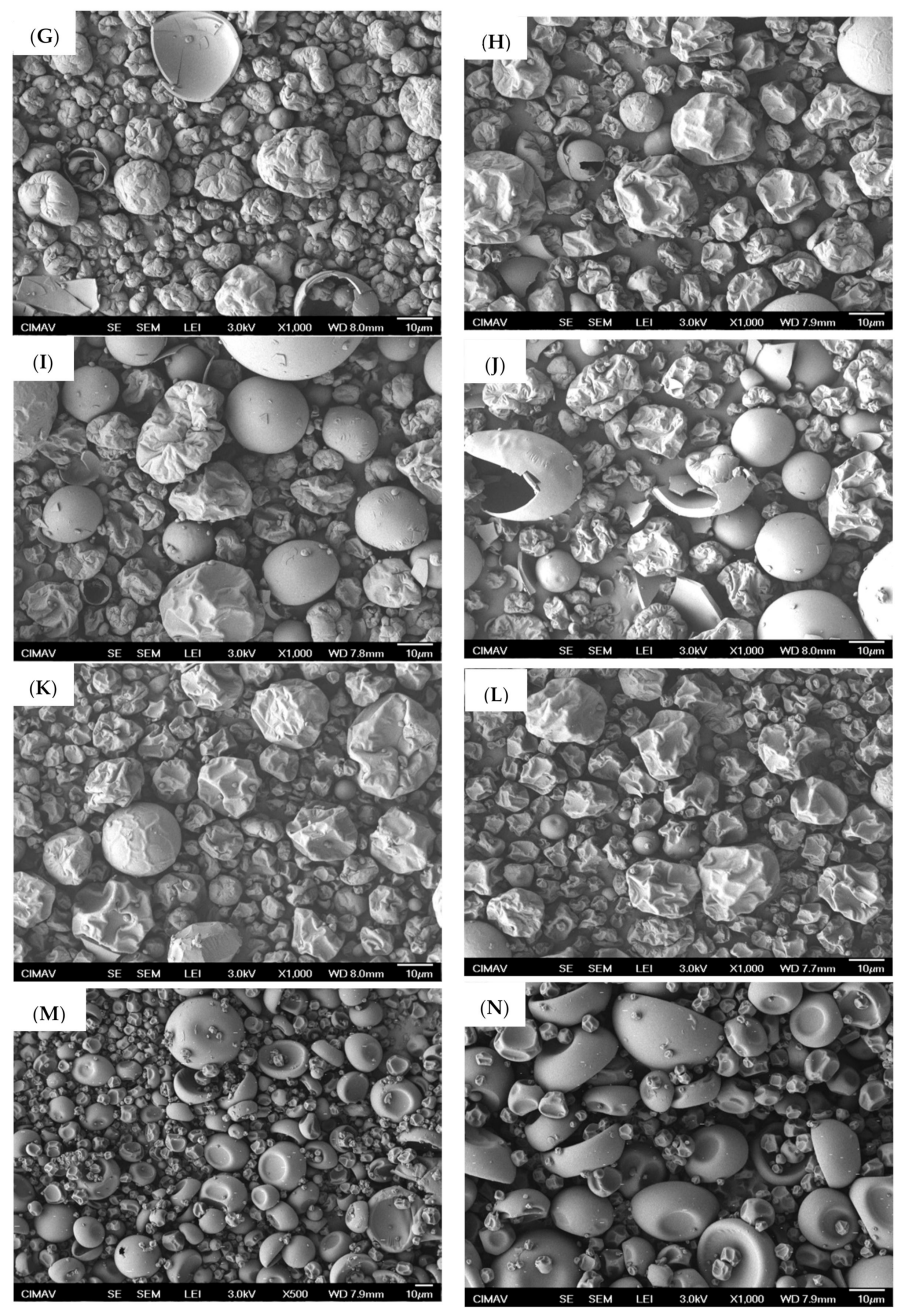
SEM micrographs of the particle morphology of the BJ-MX powders obtained under different drying conditions (**A**–**L**), and the MX employed as blank (**M**,**N**). Micrograph identification: (**A**) BS220[7.5], (**B**) BF220[7.5], (**C**) BS150[7.5], (**D**) BF150[7.5], (**E**) BS220[5], (F) BF220[5], (**G**) BS150[5], (**H**) BF150[5], (**I**) BS220[10], (**J**) BF220[10], (**K**) BS150[10], (**L**) BF150[10].

Figure 5 shows SEM micrographs of the BJ-MXpowders obtained under the different drying conditions (Figure 5A–L) with MX employed as a carrier agent (Figure 5M,N). In general, powders presented a mixture of regular spherical-shape particles with smooth surfaces, and pseudo-spherical particles with irregular surfaces. In some of the samples there were observed pieces of broken hollow spherical particles. Particle size was in the range of 1–40 μm, where the spherical particles were relatively bigger than those with a pseudo-spherical morphology. According to previous reports, the morphology of powders where MX has been employed as the carrier agent is directly related to the adsorption of water rather than to the collapse of the structure [33]. In this respect, the pseudo-spherical morphology with irregular surface indicates the collapse of the structure by the rapid evaporation of the liquid phase induced by the drying temperature. The spherical morphology observed with smooth surface suggests that the structure is being conserved. On the other hand, the morphology of the blank MX particles was different from those mixed with BJ. A large amount of MX particles presented sizes bigger than 10 μm and an appearance similar to deflated spheres with a smooth surface. Although the particle morphology in spray-dried MX was dependent on factors such as (i) the molecular weight, (ii) the experimental drying conditions, and (iii) the adsorption of water, the morphology of the BJ-MX powders corresponded to well-dried particles, i.e., without minimal adsorbed water on the particle, equivalent to water activity of 0.07 [18].

**Figure 6:**
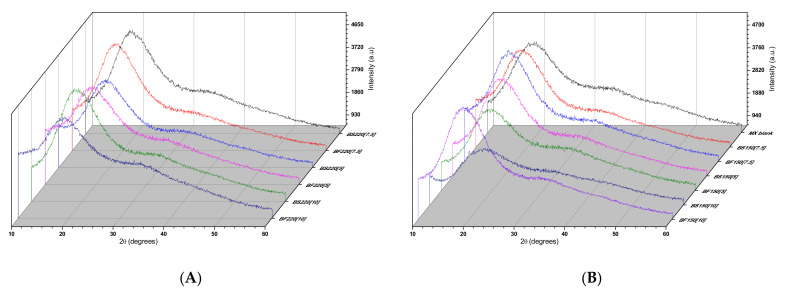
XRD diffractograms of the BJ-MX powders obtained under different drying conditions. Samples prepared at (**A**) 150 °C, and (**B**) 220 °C.

Figure 6 shows the XRD diffractograms of the BJ-MX powder samples prepared under different experimental conditions. Overall, the diffraction patterns showed a broad peak at 20° and the absence of well-defined peaks. The first observation suggested that the microstructure of the BJ-MX remained in the amorphous state under all the experimental conditions applied. In addition, since no well-defined peaks were observed, this suggested that the microencapsulated antioxidants and compounds of the BJ were not crystallizing within the maltodextrin particle, but were being preserved.

## 3. Materials and Methods

### 3.1. Materials

Broccoli florets and stalks with a greenish color and an adequate state of maturity were purchased in a local market in San Luis Potosi, Mexico. Commercial maltodextrin (MX) extracted from cornstarch was acquired from INGREDION Mexico (Guadalajara, Mexico). The dextrose equivalent (DE) of MX was 10, corresponding to a molecular weight of 1625 g/mole, and a degree of polymerization (DP) of 2–16 units of glucose [34]. Analytical grade 2,2-diphenyl-1-picrylhydrazyl (DPPH), (±)-6-hydroxy-2,5,7,8-tetramethylchromane-2-carboxylic acid (Trolox), gallic acid, sodium carbonate (Na_2_CO_3_), and Folin–Ciocalteu reagent were purchased from Sigma–Aldrich Chemical Co.

### 3.2. Extraction of Broccoli Floret and Stalk Juice

For the preparation of the aqueous extract, broccoli was washed first with soap and purified water. The broccoli florets and stalks were separated and cut into small chunks. Broccoli pieces were air-dried, then ground in a commercial food processor. In order to inactivate the enzymatic process, the collected BJ was heated in a water bath at 75 °C for 5 min, then centrifuged at 10,000 rpm for 10 min at 4 °C to remove the fiber from the liquid. The supernatant was filtered in vacuum, and the clarified juice was transferred to a plastic bottle.

### 3.3. Preparation of Spray-Dried Powders

Spray drying was employed in the preparation of the broccoli powders. The spray-drying conditions were slightly modified from those reported by Vazquez–Maldonado et al. [35]. The preparation of feeding solutions consisted of mixing 20 g of maltodextrin with 200 mL of broccoli juice. Microencapsulation was carried out in a Mini Spray Dryer B290 (BÜCHI, Labortechnik AG, Flawil, Switzerland) under the following operating conditions: feed temperature of 40 °C, feeding flow of 7 cm^3^/min, hot airflow of 28 m^3^/h, aspiration of 70%, and pressure of 1.5 bar. The inlet temperatures were varied between 150 and 220 °C. This wide range of temperatures was selected to avoid the collapse of the microstructure and other unwanted characteristics such as stickiness and agglomeration of the powders. The obtained powders were weighted and labeled according to Table 1. Powders were individually placed in airtight containers and stored in darkness at 4 °C.

### 3.4. Antioxidant Activity

The antioxidant extracts were prepared by dissolving 500 mg of BJ-MX powders in a solution of methanol and distilled water in a ratio of 7:3, respectively. The extraction was conducted under constant stirring for 2 h. After this period, the extracts were centrifuged, filtered, and stored at 4 °C in amber bottles for protection from light until the analysis of the antioxidant activity and phenolic compounds [36]. Triplicate extracts were prepared.

The extract samples were measured in terms of hydrogen-donating or radical-scavenging ability using the stable DPPH radical [37]. Briefly, the reaction mixture contained 100 mL of the extract and 3.9 mL of DPPH. The absorbance of the reaction mixture was measured at 515 nm against a blank sample containing only methanol. The results were expressed in terms of the mass of Trolox per mass of dry powder juice (mg of Trolox/g BJ-MX), and the full equivalence values were calculated using the standard curve of Trolox. 

### 3.5. Determination of Total Phenolic Compounds

The total phenolic content (TPC) of compounds in the extracts was determined via the Folin–Ciocalteu method, using gallic acid as the standard. The reaction was carried out with 0.5 mL of the extract, 2.5 mL of Folin–Ciocalteu reagent diluted (1:10) in distilled water, and 2 mL of a 4% solution of Na_2_CO_3_. The absorbance was measured at 740 nm. The TPC was expressed as the gallic acid equivalent (GAE) per mass of dry BJ-MX (mg of gallic acid equivalent (GAE)/g of BJ-MX) [38].

### 3.6. Thermal Analysis

#### 3.6.1. MDSC

A modulated differential scanning calorimeter (MDSC) Q200 (TA Instruments, USA) equipped with an RCS90 cooling system was employed for determining the glass transition (Tg). The instrument was calibrated with indium for melting temperature and enthalpy, while sapphire was used as the standard for heat capacity (Cp). Samples of about 10 mg were encapsulated in Tzero^®^ aluminum pans. Thermograms were acquired at a temperature range of −50 to 250 °C, with a modulation period of 40 s and amplitude of 1.5 °C. Each experiment was repeated three times.

#### 3.6.2. TGA-DSC-SDT

Thermogravimetric (TGA) and differential scanning calorimetry (DSC) analyses were carried out in a simultaneous TGA-DSC SDT Q600 (TA Instruments, New Castle, DE, USA). For the DSC, baseline was calibrated with indium (melting temperature of 156.6 °C and melting enthalpy of 28.47 J/g). Samples of 10 mg were encapsulated in standard aluminum pans. Thermograms were recorded at a heating rate of 5 °C/min over a range of 25–400 °C using Universal Analysis 2000© software.

### 3.7. Physicochemical Characterization

#### 3.7.1. Scanning Electron Microscopy

Morphological characterization was conducted using a scanning electron microscope (SEM) (JEOL JSM-7401F) operated at an accelerating voltage of 3 kV. Powder samples were first dispersed on graphite conductive tape, then covered with a thin layer of gold nanoparticles by means of sputtering to reduce charging effects (Denton Desk II sputter coater, Denton, TX, USA).

#### 3.7.2. X-ray Diffraction

Microstructural characterization was determined by x–ray diffraction (XRD) analysis in an D8 Advance ECO diffractometer (Bruker, Karlsruhe, Germany) equipped with Cu-Kα radiation (*l* = 1.5406 Å) operated at 45 kV, 40 mA and a X’Celerator detector in a Bragg-Brentano geometry. Scans were performed in the 2θ range of 10–100°, with step size of 0.016° and 20 s per step.

### 3.8. Statistical Analysis

All experiments were performed in triplicate, reporting mean values and standard deviations. One-way analysis of variance (ANOVA) was performed to establish a significance level of 0.05, and the Tukey’s honestly significant difference (HSD) post hoc test was used to determine the difference between the means. The statistical analyses were conducted using the IBM SPSS statistics version 21.0 software (SPSS Inc., Chicago, IL, USA).

## 4. Conclusions

Broccoli juice (BJ) was spray-dried with the aid of low molecular-weight maltodextrin (MX) as carrier agent. Several experimental settings were tested in order to find the optimal conditions of drying temperature, MX concentration, and source of broccoli. In all cases, a well-dried powder was obtained with a yellowish appearance and a strong smell of broccoli. Microstructurally, there were slight differences, mainly in the morphology of the micro particles. The thermal characterization showed some differences in the Tg of the powders obtained at 150 and 220 °C, suggesting that powders may be stored at temperatures below 50 °C. The influence of the processing conditions was mainly observed on the total phenolic content and the antioxidant activity. The effect of the drying temperature and the content of MX was similar in both tests, and the tendency was to decrease with the content of MX and with the decrease in the drying temperature. However, the source of broccoli showed a higher TPC value in BJ-MX powders when using the floret juice, while the antioxidant activity of BJ-MX powders was higher when testing the stalk juice. These observations suggested that water-soluble phenolic compounds and vitamin C are preferentially located in the florets, while antioxidant compounds are preserved better in the broccoli stalk juice-MX powders. This work demonstrated that BJ was suitable for spray drying, and that a byproduct such as broccoli stalk juice may be employed as a potential source of antioxidants.

## Figures and Tables

**Table 1:** Experimental design employed to determine the optimal drying condition of BJ-MX mixtures.

Run	MX Concentration (%)	Inlet Temperature (°C)	Broccoli Juice Source	Identification
1	7.5	220	Stalk	BS220-[7.5]
2	7.5	220	Floret	BF220-[7.5]
3	7.5	150	Stalk	BS150-[7.5]
4	7.5	150	Floret	BF150-[7.5]
5	5.0	220	Stalk	BS220-[5.0]
6	5.0	220	Floret	BF220-[5.0]
7	5.0	150	Stalk	BS150-[5.0]
8	5.0	150	Floret	BF150-[5.0]
9	10.0	220	Stalk	BS220-[10]
10	10.0	220	Floret	BF220-[10]
11	10.0	150	Stalk	BS150-[10]
12	10.0	150	Floret	BS150-[10]

## Data Availability

Data is contained within the article and Supplementary Material.

## Supplementary Materials

Spray dried vegetable juices (Table S1) and Broccoli bioactive compounds (Table S2). References [39,40,41,42] are cited in the Supplementary Materials.

## Sample Availability

Samples of the compounds are not available from the authors.
