# Supplementary material for: Physicochemical Properties and Antioxidant Activity of Spray-Dry Broccoli (Brassica oleracea var Italica) Stalk and Floret Juice Powders
Source: Molecules. 2021 Mar 31;26(7):1973. doi: 10.3390/molecules26071973 (PMC8036675; doi:10.3390/molecules26071973)
Supplement: Supplementary file 1 [file molecules-26-01973-s001.pdf]

Table S1. Spray dried vegetable juices.

Sample	Carrier	Concentration	Inlet temperature (°C)	Antioxidant Activity and TPC	Reference
Kale clorophill extract	Whey Protein Isolate	15:100 WPI:H_20<br><br>0, 5, 10 y 20% Kale clorophill	120, 150, and 180	If the inlet air drying temperature increased, the DPPH scavenging activity of WPI increased gradually from 34.06% to 53.12%. This result showed that the antioxidant capacity of WPI obtained at higher inlet air drying temperature (180 °C) was significantly better than the sample obtained at lower inlet air drying temperatures (120 and 150 °C). Chlorophyl was preserve at 120 C and 150 C. The WPI partially denatured to ex- pose more electron-donating groups such as sulfur-containing amino acids, cysteine and methionine.	[39]
Bell pepper juice	Maltodextrin (D.E. 17-19)	1:2 relationship Maltodextrin/red pepper juice	120	TPC = 2535 mg GAE/100 g DW	[15]
Artichoke leaf extract	Monohydrate α-Lactose	1:2 relationship (lactose:artichoke extract)<br><br>2.0, 2.5, 3.0 %	55, 65, and 75	TPA= 13.6 mg GAE/g DW and 18.44 % DPPH inhibition<br>In the vacuum pressure of 20 kPa, total phenol content and antioxidant capacity increased due to the lack of oxygen inside the chamber and oxidation reaction reduction of extract compounds.	[40]
Carrot – celery juice and 2:1	Maltodextrin (16.5 – 19.5)	0.7, 0.8 and 0.9 w/w	120, 145, and 175	The β-carotene content in the powder varied from 83.52 to 127.32 mg/100g. The results show that with increasing inlet air temperature, β-carotene contents were extremely decreased. Although maltodextrin has a covering and protecting role for sensitive natural pigments, the results showed that by increasing the maltodextrin concentration, the β-carotene content of the powder was decreased because of reduced carotenoid con- centration. By increasing the feed flow rate, powder particle size was increased and lower surface to volume ratio of large particles leads to more β-carotene retention in the carrot– celery powder.	[41]
Eggplant peel juice	Maltodextrin (DE: 18-20 and DP: 2-20 units)<br><br>Arabig Gum<br><br>Mixture MD/AG	10% w/v Carrier/egg plant solution	140 and 170	Total phenolic content (TPC) is one of the most important indices for evaluating the antioxidant capacity of various plant extracts.<br>We found that TPC of EPE was between 4.57 and 5.22 mg/g powder. the highest amount of TPC was related to MD powders (at 170°C). But, partial or complete replacement of MD with GA re- duced TPC (P b 0.05). When comparing to other plant extracts, microenncreasing the inlet temperature, respectively. But, Tolun et al. [11] reported the polyphenol content of grape pomace extract powder decreased when temperature increased from 120 to 160°C then a reverse tendency found in phenolic at 180 °C. They explained the polymerization of polyphenols at high temperatures as a result of an increase in TPC of the samples Nio creasing the inlet temperature, respec- tively. But,	[27]

				Tolun et al. [11] reported the polyphenol content of grape pomace extract powder decreased when temperature increased from 120 to 160°C then a reverse tendency found in phenolic at 180 °C. They explained the polymerization of polyphenols at high temperatures as a result of an increase in TPC of the samples. DPPH free radical inhibition activity for all samples varied between 55.48 and 73.38%. Powders produced with MD showed a higher antioxidant activ- ity than GA. Also, the highest ABTS+ radical scavenging activities (90.54%) and TEAC values (2.45 mM) was observed for samples pre- pared by MD at 170 °C. Results of these tests also showed a higher ability of MD compared to GA in the production of powders with a high antioxidant activity.	

Table S2. Broccoli bioactive compounds.

Variety	Part	Total Phenolics	Antioxidant Activity	Findings	Referen ce
Marathon<br>Nubia<br>Viola	Stalks<br>Leafs	TPC by HPLC (mg GAE/g dw)<br>Marathon: 9.78<br>Nubia: 8.127<br>Viola: 11.74<br><br>Clorogenic acid Derivatives<br>Marathon: 8.63<br>Nubia: 6.56<br>Viola: 9.66<br><br>Sinapic acid derivtives<br>Marathon: 8.63<br>Nubia: 6.56<br>Viola: 9.66<br><br>Vitamin C<br>Marathon: 3.635<br>Nubia: 3.350<br>Viola: 2.295	By DPPH (mM TE/g dw)<br>Marathon: 12.046<br>Nubia: 3.048<br>Viola: 7.289	The total concentration of phenolic compounds in the broccoli leaves was almost 10 times higher than in the stalks. Among these phenolics compunds, the hydroxycinnamic acids and flavonoids in the stalks of the 3 cultivars were in a very low concentration range. While, the concentration of the nutrient vitamin C, an important part of the antioxidant machinery of plant tissues, varied significantly among organs and cultivars, confirming previous data.<br>The DPPH· assay has been used widely to characterize the antioxidant capacity of Brassica spp. Vegetables. The analysis of extracts of the broccoli by-products showed a higher capacity for scavenging DPPH· in leaves than in the stalks. The antioxidant capacity of broccoli extracts could be attributable to their "natural antioxidants" (Heimler and others 2006; Podsedek 2007). Although vitamin C contributes to the antioxidant profile of many vegetables in broccoli by-products it was correlated inversely to the radical-scavenging activity (Figure 1). In this sense, other molecules could contribute to this radical-scavenging since broccoli phenolics were correlated strongly to the antioxidant DPPH· test results.	[20]
15 commercial varieties from USA	Florets<br>leaves	NA	By DPPH ≈1.5 – 14 mg AEAC g-1	The flavonols kaempferol and quercetin have gained increasing interests due to their health-promoting properties. Broccoli is known to accumulate these two flavonols. Thus, this attribute along with its multiple health-benefit nutrients and phytonutrients makes broccoli an excellent functional food. In this study, 15 broccoli accessions consisted	[32]

			Total Flavonol 31.1 to 103.9 μg RE g-1 FW	of both landraces and improved entries were examined for their flavonol levels and antioxidant associated attributes. The total antioxidant capacity as measured by DPPH and FRAP showed no great differences in both leaves and florets among these broccoli accessions, kaempferol and total flavonoids were generally consistent with the antioxidant capacity and activities of antioxidant enzymes as revealed by Pearson correlation and PCA. The antioxidant capacity was to some extent correlated with flavonoids and kaempferol, implying that total flavonoid and kaempferol contribute partly and the antioxidant capacity is a composite result of complex factors. The high level of total flavonoids does not necessarily reflect high kaempferol or quercetin content in a particular variety. In florets, positive correlation was observed between anthocyanins and FRAP, and between total flavonoids, DPPH, APX, and SOD ($p < 0.05$).	
Brassica olearacea from Nagano	Florets Stems	By Folin-Ciocalteu Assay F: 34.5 ± 1.0 mg GAE/100g FW S: 4.5 ± 0.2 mg GAE/100g FW<br><br>Ascorbic Acid F: 103 ± 3.5 mg/100g FW S: 124 ± 3.8 mg/100g FW	By DPPH analysis Total antioxidants F: 60.5 ± 2.5 % S: 62.8 ± 2.5 %<br><br>Phenolic Antioxidant s F: 60.5 ± 2.5 % S: 62.8 ± 2.5 %	In the present study, broccoli florets were found to contain 34.5 mg/100 g FW. Less than that reported by Leja et al. (2001). The difference could be explained by removal of interfering components, such as ascorbic acid, from the extract using a reverse phase Sep-Pak C18 column in the present study. Some re- ducing components, such as ascorbic acid, capable of being oxidized by the Folin–Ciocalteu reagents of the total phenolic assay, yield the reduced (coloured) forms of the reagents and appear as phenolics.The different varieties used might also cause differences. The results indicate that phenolics, ascorbic acid, and carotenoids are dis- tributed differently in broccoli floret and stem. The results obtained in the present study showed that the content of ascorbic acid declined dramatically during both conventional and microwave cooking. This indicates that cooking affects retention of ascorbic acid in the tissues. Free radical-scavenging is one of the known mechanisms by which antioxidants inhibit lipid oxidation caused by free radicals. Broccoli contained flavonol glycosides and hydroxycinnamic acids (Hertog et al., 1992; Plumb et al., 1997; Price et al., 1997, 1998). Though antioxidant activity in phenolic extracts only accounted for 20.0% and 2.7% of total antioxidant activity in florets and stems,	[11]
Brassica olearacea	Not expecified	Folin-Ciocalteu Assay (mg CE/g dw) Acetone: 5.2 ± 0.7 Methanol: 4.9 ± 0.7 Water: 4.5 ±1.2<br><br>Total Flavonoids (mg RE/ g dw) Acetone: 1.6 ± 0.6	DPPH Assay (mM TE/g dw) Acetone: 6.0 ± 2.6 Methanol: 11.5 ± 2.8 Water: 4.8 ±0.4	Flavonoid compounds in broccoli include two major compounds (quercetin 3-O-sophoroside and kaempferol 3-O-sophoroside) and three minor components (isoquercitrin, kaempferol 3-O-glucoside and a kaempferol diglucoside). The contents of quercetin and kaempferol sophorosides in raw broccoli florets were 65 and 166 mg/kg fresh weight, respectively. From the present experiment, methanol and acetone extracts had higher antioxidant activity than water extracts possibly because flavonoids are major antioxidants in both vegetables and are more soluble in methanol and acetone than water. Thus water	[42]

		Methanol: 0.9 ± 0.4<br>Water: 0.4 ± 0.1		alone is not a good solvent to extract the antioxidants of asparagus or broccoli.	
Broccoli Lord cultivar	Florets	TPC by Folin-Ciocalteu Assay<br>8.863 mg/g dw	NA	The content of total polyphenols in fresh broccoli 886.3 mg/100 g dry weight (109.9 mg/100 g fresh weight), determined in this study. Fresh broccoli contains 681.2 mg/100 g dry weight (84.5 mg/100 g fresh weight) of vitamin C. The content of vitamin C in edible parts of fresh broccoli, depend- ing on variety, may vary from 43.2 to 146.4 mg/100 g of fresh weight (Vallejo et al. 2003). Based on available biochemical, clinical, and epidemiological studies, the current recommended daily acceptance (RDA) for ascorbic acid is suggested to be 100–120 mg day®1 to achieve cellular saturation and optimum risk reduction of heart diseases, stroke and cancer in healthy individuals	[28]

References

11. Zhang, D.; Hamauzu, Y. Phenolics, ascorbic acid, carotenoids and antioxidant activity of broccoli and their changes during conventional and microwave cooking. *Food Chem.* **2004**, *88*, 503–509, doi:10.1016/j.foodchem.2004.01.065.
15. Rybak, K.; Samborska, K.; Jedlinska, A.; Parniakov, O.; Nowacka, M.; Witrowa-Rajchert, D.; Wiktor, A. The impact of pulsed electric field pretreatment of bell pepper on the selected properties of spray dried juice. *Innov. Food Sci. Emerg. Technol.* **2020**, *65*, 102446, doi:10.1016/j.ifset.2020.102446.
20. Domínguez-Perles, R.; Martínez-Ballesta, M.C.; Carvajal, M.; García-Viguera, C.; Moreno, D. Broccoli-derived by-products - a promising source of bioactive ingredients. *J. Food Sci.* **2010**, 75, C383–C392, doi:10.1111/j.1750-3841.2010.01606.x.
27. Sarabandi, K.; Jafari, S.M.; Mahoonak, A.S.; Mohammadi, A. Application of gum Arabic and maltodextrin for encapsulation of eggplant peel extract as a natural antioxidant and color source. *Int. J. Biol. Macromol.* **2019**, *140*, 59–68, doi:10.1016/j.ijbiomac.2019.08.133.
28. Gliszczynska-Swiglo, A.; Ciska, E.; Pawlak-Lemanska, K.; Chmielewski, J.; Borkowski, T.; Tyrakowska, B. Changes in the content of health-promoting compounds and antioxidant activity of broccoli after domestic processing. *Food Addit. Contam.* **2006**, *23*, 1088–1098, doi:10.1080/02652030600887594.
32. Duan, Y.; Melo-Santiago, F.E.; Rodrigues dos Reis, A.; de Figueiredo, M.A.; Zhou, S.; Thannhauser, T.W.; Li, L. Genotypic variation of flavonols and antioxidant capacity in broccoli. *Food Chem.* **2021**, *338*, 127997, doi:10.1016/j.foodchem.2020.127997.
39. Zhang, Z.-H.; Peng, H.; Ma, H.; Zeng, X.-A. Effect of inlet air drying temperatures on the physicochemical properties and antioxidant activity of whey protein isolate-kale leaves chlorophyll (WPI-CH) microcapsules. *J. Food Eng.* **2019**, *245*, 149–156, doi:10.1016/j.jfoodeng.2018.10.011.
40. Namavar, S.S.; Chayjan, R.A.; Parian, J.A.; Zolfigol, M.A. A multi-objective optimization of artichoke (Cynara scolymus L.) leaves aqueous extraction dehydration through a novel spray drying approach using response surface methodology. *Iran. J. Chem. Chem. Eng.* **2018**, *37*, 221–236.
41. Movahhed, M.K.; Mohebbi, M. Spray Drying and Process Optimization of Carrot-Celery Juice. *J. Food Process. Preserv.* **2015**, *40*, 212–225, doi:10.1111/jfpp.12598.
42. Sun, T.; Powers, J.R.; Tang, J. Evaluation of the antioxidant activity of asparagus, broccoli and their juices. *Food Chem.* **2007**, *105*, 101–106, doi:10.1016/j.foodchem.2007.03.048.
